# A Review of Hot-Melt Extrusion: Process Technology to Pharmaceutical Products

**DOI:** 10.5402/2012/436763

**Published:** 2012-12-27

**Authors:** Mohammed Maniruzzaman, Joshua S. Boateng, Martin J. Snowden, Dennis Douroumis

**Affiliations:** ^1^School of Science, University of Greenwich, Central Avenue, Chatham Maritime, Chatham, Kent ME4 4TB, UK; ^2^Department of Pharmaceutical Sciences, Medway School of Science, University of Greenwich, Chatham Maritime, Kent ME4 4TB, UK

## Abstract

Over the last three decades industrial adaptability has allowed hot-melt extrusion (HME) to gain wide acceptance and has already established its place in the broad spectrum of manufacturing operations and pharmaceutical research developments. HME has already been demonstrated as a robust, novel technique to make solid dispersions in order to provide time controlled, modified, extended, and targeted drug delivery resulting in improved bioavailability as well as taste masking of bitter active pharmaceutical ingredients (APIs). This paper reviews the innumerable benefits of HME, based on a holistic perspective of the equipment, processing technologies to the materials, novel formulation design and developments, and its varied applications in oral drug delivery systems.

## 1. Introduction

 To date HME has emerged as a novel processing technology in developing molecular dispersions of active pharmaceutical ingredients (APIs) into various polymer or/and lipid matrices which has led this technique to demonstrate time controlled, modified, extended, and targeted drug delivery [1–4]. HME has now provided opportunity for use of materials in order to mask the bitter taste of active substances. Since the industrial application of the extrusion process back in the 1930's, HME has received considerable attention from both the pharmaceutical industry and academia in a range of applications for pharmaceutical dosage forms, such as tablets, capsules, films, and implants for drug delivery via oral, transdermal, and transmucosal routes [5]. This makes HME an excellent alternative to other conventionally available techniques such as roll spinning and spray drying. In addition to being a proven manufacturing process, HME meets the goal of the US Food and Drug Administration's (FDA) process analytical technology (PAT) scheme for designing, analyzing, and controlling the manufacturing process via quality control measurements during active extrusion process [6]. In this chapter, the hot-melt extrusion technique is reviewed based on a holistic perspective of its various components, processing technologies, and the materials and novel formulation design and developments in its varied applications in oral drug delivery systems.

## 2. Process Technology of Hot-Melt Extrusion (HME)

**Figure 1:**
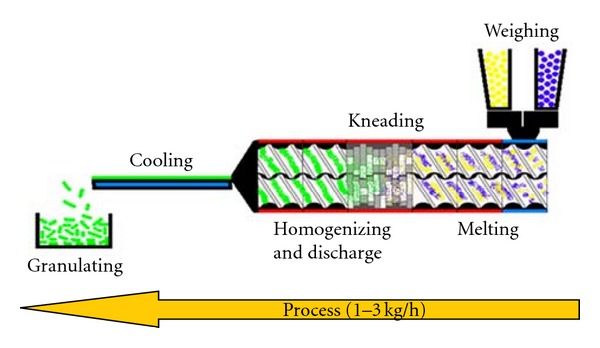
Schematic diagram of the HME process [12].

Hot-melt extrusion technique was first invented for the manufacturing of lead pipes at the end of the eighteenth century [7]. Since then, it has been used in the plastic, rubber, and food manufacturing industry to produce items ranging from pipes to sheets and bags. With the advent of high throughput screening, currently more than half of all plastic products including bags, sheets, and pipes are manufactured my HME and therefore various polymers have been used to melt and form different shapes for a variety of industrial and domestic applications. The technology (HME) has proven to be a robust method of producing numerous drug delivery systems and therefore it has been found to be useful in the pharmaceutical industry as well [8]. Extrusion is the process of pumping raw materials at elevated controlled temperature and pressure through a heated barrel into a product of uniform shape and density [9]. Breitenbach first introduced the development of melt extrusion process in pharmaceutical manufacturing operations [10]; however, Follonier and his coworkers first examined the hot-melt technology to manufacture sustained release polymer-based pellets of various freely soluble drugs [11]. HME involves the compaction and conversion of blends from a powder or a granular mix into a product of uniform shape [9]. During this process, polymers are melted and formed into products of different shapes and sizes such as plastic bags, sheets, and pipes by forcing polymeric components and active substances including any additives or plasticisers through an orifice or die under controlled temperature, pressure, feeding rate, and screw speed [9, 12]. However, the theoretical approach to understanding the melt extrusion process (Figure 1) can be summarized by classifying the whole procedure of HME compaction into the following [13]:

1. feeding of the extruder through a hopper,
2. mixing, grinding, reducing the particle size, venting, and kneading,
3. flow through the die, and
4. extrusion from the die and further downstream processing.

The extruder generally consists of one or two rotating screws (either corotating or counter rotating) inside a stationary cylindrical barrel. The barrel is often manufactured in sections in order to shorten the residence time of molten materials. The sectioned parts of the barrel are then bolted or clamped together. An end-plate die is connected to the end of the barrel which is determined according to the shape of the extruded materials.

## 3. Single-Screw and Twin-Screw Extruder

**Figure 2:**
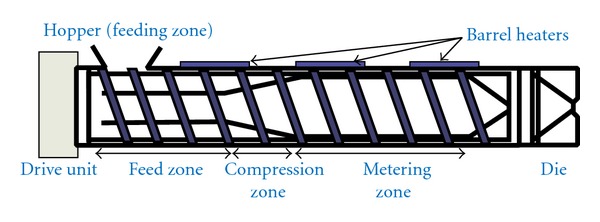
Schematic diagram of a single-screw extruder [10].

 A single-screw extruder consists of one rotating screw positioned inside a stationary barrel at the most fundamental level. In the more advanced twin-screw systems, extrusion of materials is performed by either a corotating or counter-rotating screw configuration [9]. Irrespective of type and complexity of the function and process, the extruder must be capable of rotating the screw at a selected predetermined speed while compensating for the torque and shear generated from both the material being extruded and the screws being used. However, regardless of the size and type of the screw inside the stationary barrel a typical extrusion set up consists of a motor which acts as a drive unit, an extrusion barrel, a rotating screw, and an extrusion die [13]. A central electronic control unit is connected to the extrusion unit in order to control the process parameters such as screw speed, temperature, and therefore pressure [14]. This electronic control unit acts as a monitoring device as well. The typical length diameter ratios (L/D) of screws positioned inside the stationary barrel are another important characteristic to consider whether the extrusion equipment is a single-screw or twin-screw extruder. The L/D of the screw either in a single-screw extruder or a twin-screw extruder typically ranges from 20 to 40 : 1 (mm). In case of the application of pilot plant extruders the diameters of the screws significantly ranges from 18 to 30 mm. In pharmaceutical scale up, the production machines are much larger with diameters typically exceeding 50–60 mm [15]. In addition, the dimensions of a screw change over the length of the barrel. In the most advanced processing equipment for extrusion, the screws could be separated by clamps or be extended in proportion to the length of the barrel itself. A basic single-screw extruder consists of three discrete zones: feed zone, compression, and a metering zone (Figure 2). Under the compression zone which is basically known as processing zone could be accompanied by few other steps such as mixing, kneading, and venting [13, 15].

**Figure 3:**
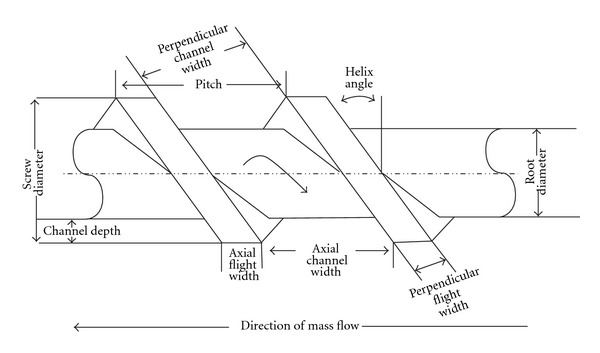
Screw geometry (extrusion) [9].

 The depth along with the pitch of the screw flights (both perpendicular and axial) differ within each zone, generating dissimilar pressures along the screw length (Figure 3). Normally the pressure within the feed zone is very low in order to allow for consistent feeding from the hopper and gentle mixing of API, polymers, and other excipients and therefore the screw flight depth and pitch are kept larger than that of other zones. At this stage of the process the pressure within the extruder is very low which subsequently gets increased in the compression zone. This process results in a gradual increase in pressure along the length of the compression zone, which effectively imparts a high degree of mixing and compression to the material (by decreasing the screw pitch and/or the flight depth) [9, 15]. Moreover the major aim of the compression zone is not only to homogenize but also compress the extrudate to ensure the molten material reaches the final section of the barrel (metering zone) in a form appropriate for processing. Finally the final section which is known as the metering zone stabilizes the effervescent flow of the matrix and ensures the extruded product has a uniform thickness, shape, and size. A constant and steady uniform screw flight depth and pitch helps to maintain continuous high pressure ensuring a uniform delivery rate of extrudates through the extrusion die and hence a uniform extruded product.

 In addition to the above-mentioned systems, downstream auxiliary equipment for cooling, cutting, and collecting the finished product is also typically employed. Mass flow feeders to accurately meter materials into the feed hopper, pelletizers, spheronizer, roller/calendaring device in order to produce continuous films, and process analytical technology such as near infrared (NIR) and Raman, ultrasound, and DSC systems are also options. Throughout the whole process, the temperature in all zones is normally controlled by electrical heating bands and monitored by thermocouples. 

**Figure 4:**
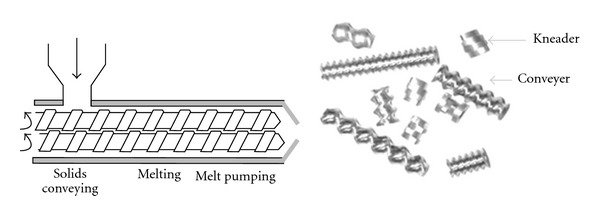
A twin-screw extruder and screws [9].

 The single-screw extrusion system is simple and offers lots of advantages but still does not acquire the mixing capability of a twin-screw machine and therefore is not the preferred approach for the production of most pharmaceutical formulations. Moreover, a twin-screw extruder offers much greater versatility (process manipulation and optimisation) in accommodating a wider range of pharmaceutical formulations making this setup much more constructive. The rotation of the screws inside the extruder barrel may either be corotating (same direction) or counter-rotating (opposite direction), both directions being equivalent from a processing perspective (Figure 4). A greater degree of conveying and much shorter residence times are achievable with an intermeshing setup. Furthermore, the use of reverse-conveying and forward-conveying elements, kneading blocks, and other intricate designs as a means of improving or controlling the level of mixing required can help the configuration of the screws themselves to be varied [16]. 

## 4. Advantages and Disadvantages of HME 

 HME offers several advantages over conventionally available pharmaceutical processing techniques including (a) increased solubility and bioavailability of water insoluble compounds; (b) solvent-free nonambient process; (c) economical process with reduced production time, fewer processing steps, and a continuous operation; (d) capabilities of sustained, modified, and targeted release; (e) better content uniformity in extrudates; (f) no requirements for the compressibility of active ingredients; (g) uniform dispersion of fine particles; (h) good stability at changing pH and moisture levels and safe application in humans; (i) reduced number of unit operations and production of a wide range of performance dosage forms (j) a range of screw geometries [17–21].

However, HME has some disadvantages as well. The main drawbacks of HME include thermal process (drug/polymer stability), use of a limited number of polymers, high flow properties of polymers, and excipients required and not suitable for relatively high heat sensitive molecules such as microbial species and proteins [20, 21].

## 5. Overall Applications of HME 

 Extrusion technology is one of the most important fabrication processes in the plastic and rubber industries. Products made from melt extruded polymers range from pipes to hoses through to the insulated wires, cables, rubber sheeting, and polystyrene tiles. Plastics that are commonly processed by HME technique include acrylics and cellulosics, polyethylene, poly propylene, polystyrene, and vinyl plastics [9, 22]. In the food industry, extrusion has been utilized for pasta production with a widely used multitalented technique combining cooking and extrusion in a self-styled extrusion cooker [23]. In the animal feed industry and veterinary science, extrusion is commonly applied as a means of producing pelletized feeds, implants, or injection moulding [24]. HME has successfully been applied in the formulation of fast dispersing PVP melt extrudates of poorly soluble active agents as solid molecular dispersions in the crop protection field [25].

 HME technology has already achieved a strong place in the pharmaceutical industry and academia due to several advantages over traditional processing methods such as roll spinning and grinding [18]. In addition to being an efficient manufacturing process, HME enhances the quality and efficacy of manufactured products and therefore over the past few years HME has emerged as a novel technique in pharmaceutical applications [15, 26]. The main use of HME is to disperse active pharmaceutical ingredients (APIs) in a matrix at the molecular level, thus forming solid solutions [27]. In the pharmaceutical industry, HME has been used for various applications, such as (i) enhancing the dissolution rate and bioavailability of poorly soluble drugs by forming a solid dispersion or solid solution, (ii) controlling or modifying the release of the drug, (iii) taste masking of bitter APIs, and (iv) formulation of various thin films [28].

 The bioavailability of an active ingredient is controlled by its aqueous solubility. Therefore, increasing the solubility of water insoluble drugs is still a real challenge in the formulation development process [27]. Due to the advent of high throughput screening (HTS) in the drug discovery process, the resultant compounds are often high molecular weight and highly lipophilic and therefore exhibit poor solubility [29]. Scientists have already tried to address solubility issues by various pharmaceutical interventions. Among the many methods available to improve solubility and dissolution rate, preparation of solid dispersions and solid solutions has gained vast attention. For that reason, HME has been successfully applied to prepare solid molecular dispersion of APIs into different hydrophilic polymer matrices [27, 29].

## 6. Applications of HME in Pharmaceutical Research

 Despite the fact that initial research developments have focused on the effects of formulation and processing variables on the properties of the final dosage forms, [9, 30–33] more recent investigations have focused on the use of HME as a novel manufacturing technology of solid molecular dispersions through the development of minimatrices, taste masked formulations, and also sustained release formulations as well as paediatric formulations [27, 34]. Early work by De Brabander et al. (2000) described the preparation of matrix minitablets which was followed by further investigations into the properties of sustained release minimatrices manufactured from ethyl cellulose, HPMC, and ibuprofen [35, 36]. Extruded minitablets showed minimised risk of dose dumping, reduced inter- and intrasubject variability. Very recently, Roblegg et al. reported the development of retarded release pellets using vegetable calcium stearate (CaSt) as a thermoplastic excipient processed through HME, where pellets with a drug loading of 20% paracetamol released only 11.54% of the drug after 8 hours due to the significant densification of the pellets. As expected, the drug release was influenced by the pellet size and the drug loading [37]. A microbicide intravaginal ring (IVRs) IVR was prepared and developed from polyether urethane (PU) elastomers for the sustained delivery of UC781 (a highly potent nonnucleoside reverse transcriptase inhibitor of HIV-1). PU IVRs containing UC781 were fabricated using a hot-melt extrusion process [38].

Moreover, a fourfold increase in the availability of propanolol in the systemic circulation was observed when the HME formulation was compared with a commercially available formulation (Inderal). Over the last five years HME has been used largely to manufacture granules, pellets, immediate and modified release tablets, transmucosal/transdermal films, and implantable reservoir devices [3, 4, 9, 33]. For instance, with respect to drug administration through the oral route, molecular solid dispersions of nifedipine [39], nimodipine [29], and itraconazole [40–42] have been successfully produced using HME technology. Amorphous indomethacin dispersions have been manufactured using pharmaceutically acceptable hydrophilic polymers by using HME technology [27, 43, 44]. 

Furthermore, HME research developments have driven targeted drug delivery systems including enteric matrix tablets and capsule systems over the last few years [45, 46]. Miller et al. have demonstrated the ability of HME to act as an efficient dispersive process for aggregated, fine engineering particles to improve dissolution rate properties by enhancing particles' wettability [47]. A very interesting investigation of Verreck and coworkers (2006) [48] determined the use of supercritical carbon dioxide (scCO2) as a temporary plasticiser during the manufacture of ethylcellulose through HME. A significant reduction in the processing temperature was achieved using scCO2 without any disadvantageous effects on the extrudate. Macroscopic morphology was significantly altered due to expansion of the scCO2 in the die. The use of scCO2 increased the surface area, porosity, and hygroscopicity of the final dosage forms. More recently Douroumis and coworkers used HME technique to effectively enhance the solubility of ibuprofen, indomethacin, and famotidine [27, 43].

The taste masking of bitter APIs is a major challenge especially for the development of orally disintegrating tablets (ODT). HME has been reported to be an effective technique to mask the bitter tastes of various APIs by the use of taste masking polymers that create solid dispersions to prevent bitter drugs from coming in contact with the patient's taste buds. More recently ibuprofen- and paracetamol-based taste masked formulations by HME have been reported [1, 43]. In these studies, taste masking has been achieved through intermolecular forces (e.g., hydrogen bonding) between the active substance and the polymer matrix by processing oppositely charged compounds through HME [49, 50]. Gryczke et al. [43] processed up to 40% IBU with only EPO (50%) and talc (10%) and demonstrated sufficient taste masking during the extrusion. It was found that increasing IBU concentrations enhanced drug-polymer interactions as IBU has been found to present plasticising effects compared to traditional plasticizers. The presence of a single Tg and the absence of IBU' melting endotherm confirmed the complete miscibility of IBU/EPO and the creation of a glassy solution where IBU was molecularly dispersed within EPO thus facilitating the higher dissolution rate and better taste masking efficiency. Later on the ground extruded materials were compressed in tablets and compared with commercially available Nurofen Meltlets Lemon ODTs and extruded tablets were found showing better taste masking efficiency as well as about 5-fold increased dissolution of poorly soluble IBU compared to that of Nurofen. More recently Maniruzzaman et al. [1] conducted a comparative taste masking study of extruded paracetamol (PMOL) with Eudragit EPO and cross-linked polyvinylpyrrolidone (Kollidon VA64) at different drug loadings ranging (30–60%). The taste evaluation of the developed formulations carried out by using an Astree e-tongue equipped with seven sensors and the generated data were analysed using multidimensional statistics. The data analysis showed significant suppression of the bitter taste for PMOL for both polymers.

**Figure 5:**
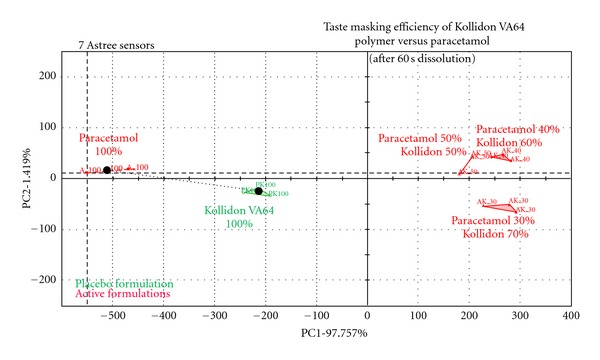
Electronic tongue “taste map”: PCA analysis of the electrode response between pure PMOL and extruded formulations with VA64 polymer after dissolution for 60 s [1].

Actually in the forgoing study taste masking of bitter PMOL was strongly dependant on the nature of the polymeric carriers and the drug loading in the final formulation. Both polymers showed excellent taste masking for active concentrations up to 50%. Additionally, *in vitro* taste analysis (taste maps) showed significant discrimination between placebo and extruded formulations (Figure 5).

The extrusion of solid lipids using twin-screw extruders was introduced for the preparation of immediate or sustained release taste-masked matrices [51]. Being divert from the polymeric extrusion the lipidic matrices offer several advantages over polymeric matrices during the extrusion process. Lipid excipients can easily be processed in 20–25°C above the room temperature and sometimes 10–15°C lower than the melting temperatures of the lipid itself; thus, extrusion does not need to be plasticizer assisted or dependent. Breitkreutz et al. successfully applied HME in taste masking of sodium benzoate for the formulation of paediatric drugs [34]. In this study the authors introduced “cold solvent-free extrusion” which utilizes different lipids in lower temperatures or even slightly above the room temperatures. In this study sodium benzoate was extruded with Precirol ATO5 (glycerol distearate), hard fat, and stearic acid in order to mask the taste of API. Mainly, the effective taste masking was achieved for at least 5 min in the buccal cavity due to the Eudragit E coating layer which prevented the release of sodium benzoate. Later on the same group processed various binary, ternary, and quaternary mixtures of powdered lipids with sodium benzoate through solvent-free cold extrusion to prepare immediate release pellets with solid lipid binders and compare them to well-known wet extrusion binders such as microcrystalline cellulose or *κ*-carrageenan. In this particular study the authors did not examine the masking efficiency of the manufactured lipid formulations which eventually made cold extrusion process questionable for the taste masking purposes. However, further evaluation of this process is a prerequisite until a considerable optimization is achieved for this method to be a viable taste masking approach. In these few studies, the effect of lipid composition and processing parameters such as the die diameter, the size of the extruded pellets, the screw speed, and the powder feeding rates on the obtained drug release patterns were thoroughly investigated. Very recently, Vaassena et al. (2012) applied solid lipid extrusion at room temperature for the taste-masked formulation development of the BCS Class II drug NXP 1210. In this study, the authors investigated powdered hard fat (Witocan 42/44 mikrofein), glycerol distearate (Precirol ato 5), and glycerol trimyristate (Dynasan 114) as lipid binders. The lipid-based formulations designed in this study was feasible for taste-masked granules or pellets containing poorly soluble drugs [52].

### 6.1. Application of HME for the Developments of Films

 However, only a handful of researchers have reported the use of hot-melt extrusion for the manufacture of films. Films can be defined as thin sheets containing one or more polymers with or without a plasticiser and may be used as a drug delivery system (device) or directly applied to facilitate a therapeutic effect as in wound dressings. Films are currently being produced mainly by solvent casting in which polymers (and excipients such as plasticisers) are dissolved in a suitable solvent until they form clear viscous solution (gel). While film preparation using the solvent-casting approach allows film uniformity, clarity, flexibility, and adjustable thickness to accommodate drug loadings they are limited by decreased elongation or elasticity and increased film tensile strength when physical aging is applied [53]. Another limitation associated with solvent cast films is the use of organic solvents for water insoluble polymers. The hazardous nature of most organic solvents and the residual solvents after drying affect the selection of the appropriate solvent [54–57] as well as complicated processing conditions and disposal of the associated waste, all of which create significant environmental concerns. As a result, alternative technologies are needed in the pharmaceutical industry to overcome some of the challenges described above. The two commonly used approaches include spray coating and hot-melt extrusion with the latter becoming increasingly popular due to the many advantages it provides. Firstly, no solvents are used and fewer processing steps are required. In fact one of the key advantages of HME is the fact that extrudates can be obtained in a single processing step making it very economical. As far as films are concerned, there is no requirement for compressing of the active ingredients together with the excipients. The melting of the polymer into the molten state, coupled with the thorough initial mixing, allows a more uniform dispersion of fine particles. Further, molecular dispersion of the drug helps to improve its bioavailability [58]. Hot-melt extruded films are produced through a simple process involving blending of appropriate amounts of relevant polymer, drug, and plasticiser into a uniform powdered mixture prior to feeding through the hopper of the preheated extruder and transferred into the heated barrel by a rotating extruder screw. Homogeneous films are obtained with thickness generally expected to be in the range less than 1 mm. Generally three main ingredients are required for successful formulation of hot-melt films, that is, film forming polymer, active ingredient, and plasticiser [59]. The latter is required to impart flexibility to the final film which ensures ease of handling and application to the site of action. Occasionally, other additives are added to affect other functionally important properties such as bioadhesive agent which ensures that the film adheres to the mucosal surface for a long enough time to allow drug absorption or action. Different polymers and drugs have been employed and reported in the literature for obtaining drug-loaded hot-melt extruded films for various indications and are summarised in Table 1. 

 Repka and coworkers have conducted extensive research on the use of HME for the manufacture of mucoadhesive buccal films. They successfully evaluated different matrix formers and additives for the processing of the blend prior to extrusion [60–63]. In an early investigation, it was found that even though films containing exclusively HPC could not be obtained, the addition of plasticizers, such as triethyl citrate, PEG 2000/8000, or acetyltributyl citrate, allowed for the manufacture of thin, flexible, and stable HPC films [64]. It has also been found that increasing the molecular weight of HPC decreased the release of drugs from hot-melt-extruded films which resulted in dissolution profiles exhibiting zero-order drug release. According to the models applied in the research, the drug release was solely determined by erosion of the buccal film [65–67].

 Development of films by HME may present future opportunities to develop gastroretentive films for prolonged drug delivery and multilayer films to modulate drug release for oral and transdermal applications. The growing market in medical devices, including incorporating drugs into devices such as biodegradable stents and drug-loaded catheters, will undoubtedly require HME manufacturing processes. These are required to be commercialised and perhaps may lead to new areas of collaboration across pharmaceutical, medical device, and biotechnology research.

## 7. Available Commercial Products Processed via HME 

 HME-related patents which have been issued for pharmaceutical systems have steadily increased since the early 1980s. So far, the USA and Germany hold approximately more than half (56%) of all issued patents for HME in the market [69]. Despite this increased interest, only a handful of commercialized HME pharmaceutical products are currently marketed. Several companies have been recognized to specialize in the use of HME as a drug delivery technology, such as PharmaForm and SOLIQS (Abbott). Recently, SOLIQS has developed a proprietary formulation which is known as Meltrex and redeveloped a protease-inhibitor combination product, Kaletra. Kaletra is mainly used for the treatment of human immunodeficiency virus (HIV) infections. The formulated, melt-extruded product was shown to have a significant enhancement in the bioavailability of active substances [70]. Furthermore, HME Kaletra tablets were shown to have significant advantages for patient compliance (i.e., reduced dosing frequency and improved stability) compared to the previous soft-gel capsule formulation as recognized by the FDA decision to fast-track approval. Additionally, Nurofen (Meltlets lemon) is available on the market as a fast dissolving tablet prepared by similar melting based technique [43]. Ibuprofen has been used as active substance in the Meltlets tablets. Moreover, SOLIQS has also developed a fast-onset ibuprofen system and a sustained-release formulation of verapamil (Isoptin SR-E) through a HME-related technology called “Calendaring” that was the first directly shaped HME product on the market. 

## 8. Conclusion

 HME has proven to be a robust method of producing numerous drug delivery systems and therefore it has been found to be useful in the pharmaceutical industry enlarging the scope to include a range of polymers and APIs that can be processed with or without plasticizers. It has also been documented that HME is a solvent-free, robust, quick, and economy-favoured manufacturing process for the production of a large variety of pharmaceutical dosage forms.

## Figures and Tables

**Table 1:** Different hot-melt extruded films comprising different polymeric materials, plasticisers, and active ingredients for various indications.

Film	Main polymer(s)	Plasticiser/additive	Main active ingredient(s)	References
1	Acrylic	Triacetin	Lidocaine	Mididoddi and Repka [68]
	Eudragit	Triethylcitrate		

2	Hydroxypropylcellulose	N/A	Ketoconazole	Prodduturi et al. [60]
	Polyethylene oxide			

3	Hydroxypropylcellulose	Polyethylene glycol 3350	Lidocaine	Repka et al. [61]
	Hydroxypropylmethylcellulose			
	Polyethylene oxide			

4	Hydroxypropylcellulose	Polyethylene glycol 400	Hydrocortisone	Repka et al. [64]

5	Hydroxypropylcellulose	Polyethylene glycol 3350	Clotrimazole	Repka et al. [58]
	Polycarbophil			

6	Polyethylene oxide	N/A	Ketoprofen	Tumuluri et al. [59]
